# Traffic-related air pollution and brain development

**DOI:** 10.3934/environsci.2015.2.353

**Published:** 2015-05-06

**Authors:** Nicholas Woodward, Caleb E. Finch, Todd E. Morgan

**Affiliations:** Davis School of Gerontology and the Dornsife College, Department of Biological Sciences, University of Southern California, 3715 McClintock Ave, Los Angeles CA 9009-0191, USA

**Keywords:** brain, body weight, prenatal, traffic-related air pollution, inflammation, PM2.5, ultrafine PM

## Abstract

Automotive traffic-related air pollution (TRP) imposes an increasing health burden with global urbanization. Gestational and early child exposure to urban TRP is associated with higher risk of autism spectrum disorders and schizophrenia, as well as low birth weight. While cardio-respiratory effects from exposure are well documented, cognitive effects are only recently becoming widely recognized. This review discusses effects of TRP on brain and cognition in human and animal studies. The mechanisms underlying these epidemiological associations are studied with rodent models of pre- and neonatal exposure to TRP, which show persisting inflammatory changes and altered adult behaviors and cognition. Some behavioral and inflammatory changes show male bias. Rodent models may identify dietary and other interventions for neuroprotection to TRP.

## 1. Introduction

Air pollution from fossil fuel combustion is increasingly recognized for its globally adverse effects on health throughout life. We focus here on traffic-related air pollution (TRP) from roadways in urban settings, for which there is strong epidemiological association with cardiovascular and pulmonary morbidity and mortality [1-4]. Moreover, TRP shows increasing evidence for impact on the brain. Thus, pre- and neonatal developmental exposure to TRP increases risk for low birth weight, and numerous cognitive detriments, including autism spectrum disorders (ASD), schizophrenia, delayed development and cognitive impairment [5-8]. The risks for these disorders are especially high during development, and pollution exposure during gestation can alter development and create lifelong deficits.

First, we discuss epidemiological data on the effects of TRP exposure during gestational development, including impaired fetal growth, as this is often associated with cognitive defects, and the effects of TRP exposure on later life cognition are reviewed. Rodent models are evaluated, as well as the relevant data produced from those models in this nascent field.

## 2. Overview of TRP

We review two broad groups of TRP: the airborne particulate matter (PM) and the vapor (gaseous) phase, with emphasis on the particulate matter component of TRP. Urban TRP PM is a complex and heterogeneous mixture that includes residues from fossil fuel combustion, organic chemicals, trace metals, nitrate, and sulfate. There are also airborne components from brake linings and the vehicular chassis, as well as roadway components and dust. The recognized size classes of airborne PM range from coarse PM (> 10 μm diameter) to microscopic classes with aerodynamic diameters less than 2.5 μm (PM_2.5_) and 0.1 μm, (PM_0.1_). For each class, primary emissions are transformed from exposure to sunlight and atmospheric ozone and nitric oxides during diurnal and seasonal cycles. While coarse PM are largely trapped by the upper airways, smaller PM can impact the brain directly from olfactory neurons in the nasal mucosa, as well as by systemic effects from the lower airways [9]. The smaller PM sizes are associated with many pathological effects of air pollution [10,11]. Although some studies lump the two smaller size classes under PM_10,_ all three categories have notable adverse effects, as well as different distributions in space and dispersion characteristics.

Of the three categories of particulate matter, PM_2.5_ (fine PM) has received the most attention, with current US EPA standards [12] of 12 μg/m^3^. The EPA has not yet addressed PM_0.1_ (UFP, or ultrafine particulates). This class of TRP warrants attention in public health because of experimental evidence for its greater cytotoxicity [13,14], potentially due to the greater penetration through cell membranes [15]. One reason UFP has not been fully appreciated is due to monitoring technology of PM based on weight and not particle number, where it is a large percentage of the total PM. We note alternate terminology of nanoscale PM, PM_0.2_, which encompasses a larger portion of the ultrafine particles, and is considered alongside UFP in this review. UFP is associated with numerous adverse health effects, and comprises the majority of all PM in combustion-derived exhaust [15]. Their small size facilitates the crossing of physiological barriers, including the blood-brain barrier and the placenta as discussed below [6,9,16].

For discussion of developmental exposure, we briefly note that adverse health effects of air pollution exposure increase with closeness to major road [10,17]. UFP was reduced by approximately 80% at a distance of 150 meters from the roadway [11]. Neither PM_2.5_ nor PM_10_ decreased substantially within 150 meters [18], and are decreased by less than 20% at a distance of 400 m vs. 50 m from a roadway [19]. The rate of dilution of UFP was correlated with increased cardiopulmonary mortality, inversely with distance from roadways [10].

## 3. Epidemiology

### 3.1. Low birth weight

Because developmental cognitive defects are often associated with fetal growth retardation, it is important that TRP can impact fetal growth. Associations between air pollution exposure in gestation and impaired fetal growth continue to emerge. In particular, PM_2.5_ is associated with low birth weight (LBW) (< 2,500 g), preterm birth, and small gestational size [20]. However, the critical period for exposure during pregnancy and threshold for these effects remain undefined. We discuss select large-scale studies of PM exposure during gestation. Also see the comprehensive review of Shah and Balkhair [20].

A large Los Angeles based study (*n* = 220,528) showed 5% greater risk of LBW from PM_2.5_ exposure, with a range of 2.4 μg/m^3^ [7]. Other studies used ultrasound to determine the gestational timing of LBW association with air pollution. The largest of these studies (17,660 pregnancies) showed the most consistent association PM_10_ exposure during days 91–120 of pregnancy, where high PM_10_ correlated with smaller abdominal circumference, heard circumference, and femur length [21]. Though this study did not find association with nitric oxide (NO_2_) exposure, other studies associated exposures of NO_2_ > 38 μg/m^3^ with reduced fetal size, femur length, and biparietal distance, even when high NO_2_ was recorded only for weeks 12–20 [22,23]. Other studies associated elevated PM_10_ exposure with preterm birth [23,24,25]. A potential mechanism underlying LBW is oxidative stress from maternal exposure during pregnancy to TRP, e.g. increased placental DNA adducts [26].

Obesity is also showing association with air pollution components, which may contribute to diabetes and the metabolic syndrome [27]. Adults (*n* = 5,228) exposed to NO_2_ showed 17% higher risk of diabetes mellitus in the top vs. lowest quintile, differing by 4 ppb [28]. There are also correlations between PM_10_ exposure and the white blood cell count, a marker for systemic inflammation [29].

### 3.2. Cognitive changes

Epidemiological studies of TRP show negative associations with adult cognition [5,30,31] and brain development [5,30,31,32]. In particular, pre- and postnatal exposure to urban TRP is correlated with autism spectrum disorders (ASD), schizophrenia, and impaired cognitive development. We briefly summarize these findings.

ASD was associated with local gradients in components of TRP, mainly PM_2.5_. Two studies utilizing the California based CHARGE (Childhood Autism Risks from Genetics and the Environment) database found about 2-fold higher odds ratio (OR), for development of ASD, when living near a freeway during the 3rd trimester, and at delivery (< 309 m defined as near, with > 1,419 m as reference group) [31,33]. Exposure during the first postnatal year was associated with 3-fold higher OR for ASD [31]. PM_2.5_ had an OR of 2.08 for gestational exposure, and 2.12 for exposure in the first year of life [31,33]. Similarly, the Nurses' Health Study, a national sample, showed an OR of 2.0 for prenatal diesel particulate exposure, top vs. bottom quintile (PM_2.5_ 4.40 vs. 0.60 μg/m^3^) [34]. Contrarily, a study of Swedish twins did not find association of TRP with ASD (PM_10_ 3.3–4.2 μg/m^3^); however, this study measured a broader size range of particles [2]. An analysis of 35 pollution components showed higher OR for ASD after exposure to methylene chloride, quinolone, and styrene, but not after diesel PM, or polycyclic aromatic hydrocarbons (PAHs) [35]. The authors noted that the control group had impairments of speech and language, which may have biased the results towards null findings.

Schizophrenia risk is also sensitive to TRP in top vs. bottom quintiles of urbanicity (population density) during gestation, but not during childhood [30]. A study of traffic volume and urbanicity (household crowding, social stressors) concluded that only traffic volume exposure at birth predicted schizophrenia (OR of 4.40 for the top vs. lowest quintile of traffic exposure) [5]. Both studies agree that only exposure during the gestational period correlated with increased risk.

TRP exposure during development is also associated with subclinical cognitive effects, including lower mental development, increased anxiety and depressive behavior, and attentional problems [32,36,37]. A Spanish national study showed decreased mental development for infants of mothers exposed during pregnancy to elevated NO_2_ and benzene [36]. Importantly for potential interventions, this association was attenuated in mothers who self-reported a high intake of antioxidant rich foods. We also note the benchmark study of Perera et al. 2003 [38] on PAH levels for Hispanics and African Americans in New York City, which was the first to utilize personal monitors for PAH levels, with greater precision than citywide measurements. Developmental measurements at birth associated high PAH (> average 3.7 ng/m^3^ in maternal blood) with a 9% decrease in birth weight, and a 2% decrease in head circumference. The OR for cognitive developmental delay, at 36 months from PAH exposure during gestation was 2.89, for the top vs. bottom quintile [37]. By age 6–7 years, individuals in the top exposure quintile were more anxious and depressed (OR 1.45), with more attentional problems (OR 1.28, top vs. bottom quintile) [32]. For DSM-IV oriented anxiety problems, the OR was a striking 4.59 [32].

## 4. Rodent models

### 4.1. Experimental approaches

Several labs have developed rodent models studying the developmental effects of TRP exposure, but no single paradigm has become widely accepted. The main findings (Tables 1–6) include effects on brain morphology, behavior, inflammatory markers, and neurotransmitters. Four experimental paradigms are currently used (Table 1): direct diesel exhaust inhalation, diesel exhaust particle (DEP) oropharyngeal aspiration, the Concentrated Air Particles delivery System (CAPS), and filter-trapped nano-sized ambient reaerosolized particles. Most studies used inhalation, while one lab used direct oropharyngeal aspiration of DEP.

#### 

##### Diesel exhaust

Pregnant mice were exposed to the whole exhaust stream from a diesel engine, diluted to concentrations ranging from 0.171–3.0 mg/m^3^. Auten et al. 2012 [47] and Bolton et al. 2012 [48] utilized a 6.4 hp direct injection single cylinder 320-cc Yanmar L70V diesel generator, operating at a constant 3600 rpm. Yokota et al. 2009 [39] used a 2369-cc diesel engine, operating at 1050 rpm. The exhaust includes volatile gaseous components, notably CO, SO_2_, and NO_2_. Unlike other exposure paradigms, these particles are not filtered by size, and retain native charges. The direct diesel exhaust paradigm is missing other real world pollutants from vehicular traffic, e.g. rubber from tire erosion, brake lining debris, and reaerosolized dust from roadways. Moreover diesel engine exhaust can represent only one type of vehicle, and the particles are being directly emitted and thus did not undergo the secondary reactions from heat and sun exposure, which develop as a function of time after emission.

#### Diesel exhaust particles (DEP)

##### Oropharyngeal Aspiration

(Auten et al. 2012 [47]) This is the only non-inhalation paradigm used in the prenatal studies. Diesel exhaust particles are collected from a single cylinder diesel engine, and then 50 μg of diesel exhaust particles (DEP) are suspended in 50 μL of PBS with 0.05% Tween-20 and delivered by oropharyngeal aspiration. Importantly, this delivery method bypasses nasal inhalation.

##### Reaersolized Inhalation

(Hougaard et al. 2008 [40]) Obtained from the National Institute of Standards and Technology, Standard Reference Material 2975, these particles are obtained from a diesel powered forklift, and are re-aerosolized for inhalation delivery [40,41]. Importantly, the re-aerosolized DEP, like the resuspended DEP for oropharyngeal aspiration, lack gaseous and volatile components. Also, because the particles are suspended in water, they are depleted of insoluble PM. Elimination of insoluble particulate matter is of special relevance, as this includes black carbon and polycyclic aromatic hydrocarbons (PAHs).

CAPS (Concentrated Ambient Particle System): Ultrafine fractioned particulate matter is concentrated next to a roadway for direct real time delivery at 10–20 times ambient concentration [42]. CAPS maintain ambient components, including gases and volatiles, and the native charges of the particles. Importantly, condensing the particles does not alter their natural size distributions, and does not amplify aggregation [13,42]. Limitations of this system are its dependence on the current traffic patterns, which fluctuate diurnally and seasonally.

Filter-trapped nano-sized PM from urban TRP: This paradigm, developed by Constantinos Sioutas at the University of Southern California [43], collects ambient air particulate matter, PM_0.2_, on the roadside next to a high traffic source with a high-volume ultrafine particle sampler on 0.2 μm pore Teflon filters. Collections are made for 4–6 weeks in the fall to encompass the range of temperatures and moisture in Southern California [44,45]. The collection is continuous and includes secondary transformations during the diurnal cycle. Besides combustion products, the sample includes reaerosolized roadway dust, and traces from brake lining and tire erosion that are < 0.2 μm. The filters are sonicated in distilled water to yield a suspension, which is stored frozen until use. The reaerosolized PM has average particle size of 60 nm at a density of 350 μg/m^3^, which is about 25× greater than ambient concentrations of that size range of particle [44,45]. The resuspension lacks ambient gases and is depleted in water insoluble organic species including PAHs and black carbon [43]. We designated these materials as nPM (nanoparticulate matter) in distinction from the size class of ambient UFP in the literature. After re-aerosolization, rodents are exposed to nPM together with ambient pollutants in the exposure room, which are 35–50% below outdoor ambient levels, while control animals have this air filtered by HEPA filters.

In summary, each experimental paradigm represents trade-offs. While DE is the most readily obtained, they are model emissions of one engine type and lack secondary atmospheric transformations of ambient pollution. The CAPS fully capture TRP for PM, gases, and volatiles, but vary diurnally and seasonally. The nPM capture diurnal variations, but the resuspension is deficient in black carbon (BC) and PAH among other water insoluble organic compounds.

### 4.2. Body weight

Epidemiological findings of air pollution on birth weight are corroborated in some rodent models (Table 2). Mice exposed to 3.0 mg/m^3^ DEP had decreased fetal weight as early as gestational day (GD) 18 (equivalent to the human third trimester) [46]. Several studies observed a weight decrease four weeks after birth in mice exposed to DEP (Table 3) [40,47]. Finally, two studies observed a reversal later in life, with increased weight at four months of age in mice [48], and at eight weeks in rats [49]. Decreased weight at weaning [40,47], combined with increased weight later in life [48,49], corresponds with previously documented rebounding in weight after a prenatal stressor [50]. Air pollution also has an additive effect on weight when exposure occurs in utero, followed by a high fat diet (HFD = 45% calories from fat) after birth (Table 4) [48,51]. The prenatal DEP + HFD treated mice showed significant weight gain over high fat diet alone, a compounding effect similar to what was observed for inflammatory responses (increased cytokines, microglial activation) [48,51]. Males in both replicates showed greater weight gain from treatment [48,51]. However, females showed 4.4× greater weight gain for the first experiment [48], and no change in the second [51]. Notably, the first experiment used diesel exhaust inhalation, while the second employed oropharyngeal aspiration of DEP, which excludes gas components. Thus, it is possible that either a species lost in the conversion to suspended diesel particles, or the inhalation delivery route caused the weight gain. The high fat postnatal group showed insulin resistance with elevated serum insulin in the males [48,51]. Females showed only a change in serum leptin, while males did not show any differences from pollution exposure.

Exposure to highly polluted ambient Beijing air (75.3 μg/m^3^) caused, worsened lipid profiles and weight gain in both rat mothers and offspring [49]. Pregnant dams had higher low-density lipoprotein (LDL), total cholesterol, triglyceride, and overall weight. Pups had increased weight at eight weeks, and worsened lipid profiles, with increased LDL, total cholesterol, and triglyceride, and decreased in HDL. Rodent models also corroborated mid-life and gestational weight effects [27,48,51,52].

### 4.3. Behavioral changes

Behavioral changes from developmental pollution exposure include cognitive and locomotor deficits (Table 2). Cognitive deficits include depressive symptoms, impaired short-term memory, and decreased response rates for fixed interval sixty-second (FI60) reward tests [53,54]. Mice exposed in utero to nPM showed increased immobility on a tail suspension test, which is a marker for depression [54]. Only males were vulnerable, with a decreased delay until first period of immobility, and 2.6× longer immobility versus control, which implicates activation of the amygdala [55,56]. Correspondingly, prenatal exposure to DEP (maternal oropharyngeal DEP aspiration) increased adult amygdala levels of monoamine neurotransmitters (dopamine, dopamine metabolites, and serotonin) [57]. However, the oropharyngeal DEP model did not alter adult forced swim performance, another marker for depression considered equivalent to tail suspension [48]. This discrepancy could be due to the different age at assessment (8 mo vs. 4 mo), or the different pollutant models.

Impaired short-term memory was observed in neonatal mice exposed to CAPS, from postnatal day (PND) 4–7 and 10–13 and assessed at 2 months of age by the novel object recognition test. In the one-hour posttest, CAPS exposed mice spent more time with the familiar versus novel object, indicative of impaired short-term memory [53]. The FI60 test, a model of impulsivity, showed decreased response and run rates, but only for males [53]. Despite smaller overall response rates, there was no significant difference in learning. In a separate experiment, conducted with the same exposure protocols, a secondary dose of pollution from PND 56–60 caused deficits in a fixed interval waiting for reward test, a classic model of impulsivity control [58]. These experiments included CAPS exposure on postnatal days 4–7 and 10–13. The sensitivity to neonatal exposure is important because neonatal rodent nervous systems are relatively less mature compared to humans [59].

Deficits in short-term memory of neonatal CAPS exposure may be mediated by glutamatergic changes. Glutamate levels in the hippocampus, which is critical for spatial learning and memory, were increased 1.26-fold in the male CAPS exposed mice [58]. Though the effect returned to baseline by eight weeks of age, the transient glutamatergic increase during development could cause persisting effects, including excitotoxicity. Detailed studies of hippocampal circuit functions, e.g. LTP, and synaptic density are needed. Increased inflammatory cytokine levels (Il-6, IL-1b, TNF-a) are also relevant to behavioral deficits through their impact on synaptic plasticity. These cytokines showed complex changes in different brain regions in mice exposed to neonatal CAPS [58,60]. For example, TNF-a modulates glutamate, and potentiates the cell to glutamatergic excitotoxicity [61], which could alter short-term memory later in life.

Locomotor deficits from prenatal exposure to pollutants include decreased spontaneous motor activity and impaired balance (Table 4) [39,48,57,62]. Intriguingly, only males have shown decreased spontaneous motor activity in studies from several labs that include ages from 5 weeks to 5 months [39,60,62]. Decreased spontaneous motor activity at age 2 months in CAPS studies was only observed when paired with a second treatment from PND 56–60 (Table 4) [53]. Balance was impaired on the rotating rod test in prenatally exposed male mice at 5 weeks [57]. These mice also had decreased latency in the cliff avoidance test [57]. The impaired performance on these two balance tests is not attributable to differences in body weight [57]; only Bolton et al. 2012 reported weight differences [51]. We note that a shift from direct exposure to diesel exhaust to the oropharyngeal DEP in the same lab did not to replicate these effects [51].

### 4.4. Gross brain morphology

Gross brain weight has not shown sensitivity to prenatal exposure to nPM [54]. However, one study of neonatal exposure to concentrated ambient TRP reported gross enlargement of the lateral ventricles, particularly in males [63]. The nPM prenatally exposed mice revealed no gross brain abnormalities, but quantitation is needed. Cerebral vasculature has just begun to receive attention in air pollution models. After maternal intranasal exposure to black carbon, young adult mice had focal induction of GFAP in astrocyte endfeet on capillary endothelia and altered arterial macrophage granules [64]. These reports point to structural changes that could underlie cognitive dysfunctions from prenatal exposure.

### 4.5. Neuronal changes

We note a major gap between the body of epidemiological evidence for TRP associations with brain development and the scant information on neuronal changes in animal models of prenatal TRP exposure. Reports on neuronal changes are scattered among different neurotransmitters, often in different brain regions, giving little cohesion of results (Table 5). Dopamine levels in the cerebral cortex illustrate the diversity. Mice exposed to prenatal diesel exhaust had lower cortical dopamine for males at 3 weeks, but no change at six weeks [57]. However, the same exposure paradigm in a different lab showed increased cortical dopamine at 5 weeks [62]. These studies used > 5-fold different levels of DEP density. The turnover of dopamine, estimated by the ratio of the catabolite DOPAC to dopamine, (DOPAC: DA) was higher in neonatally CAPS exposed male mice at two and eight weeks [58].

Neurotransmitter changes reported for adult rodent TRP exposures have not been borne out by prenatal exposures, potentially indicating different mechanisms. We observed decreased glutamate receptor 1 (GLUR1) in the hippocampus in mice exposed to nPM at age three months [43]. However, prenatal exposure did not alter hippocampus GLUR1 at eight months [54]. Neonatal exposure to DEP transiently increased hippocampal glutamate at two weeks, with return to baseline by eight weeks [58].

Cortical neurons harvested from one day old pups prenatally exposed to nPM showed impaired differentiation and neurite initiation, with fewer stage 3 neurons, compared to controls [54]. These pilot studies give a model for linking developmental exposure to alterations in neurons and glia.

### 4.6. Inflammatory changes

Inflammation may be a major mediator of maternal systemic and placental responses to air pollution exposure [60]. Systemic inflammation in the mother increases circulating inflammatory cytokines, influencing the development of the fetus, through methods such as the activation of microglia (Table 6) [48,65]. As noted above for neuronal changes, there is a need for similar protocols across labs in the investigation of inflammatory effects.

Prenatal exposure of mice to diesel exhaust rapidly increased cytokines (IL-1b, IL-6, IL-10, and TNF-a) on GD18 [47,48]. Microglial activation is suggested by increased chemokines CCL2/MCP-1 and CX3CL1/Fractalkine [48]. After CAPS neonatal exposure, proinflammatory cytokines (IL-6, IL-1b, TNF-a) were decreased at 2 weeks in males [58]. However, by eight weeks, a full month after the cessation of exposure, IL-1b and TNF-a rebounded to levels 1.4-fold above control's in midbrain [58]. Females showed a different time course, with little upregulation immediately following exposure, yet still showing delayed increases a month later. This effect was brain region specific: unlike the midbrain, the striatum had lower cytokines at the same times [58]. These sex differences in cytokine responses between different brain regions clearly show that inflammatory effects of pollution exposure must be studied in terms of brain pathways and cannot be generalized to the entire brain.

Glial inflammatory responses are detected by the astrocyte specific GFAP (intermediate filament glial fibrillary acidic protein) and the microglial marker of IBA1 (ionized calcium-binding adapter molecule 1). These responses were observed in neonatal exposures, and prenatal exposures compounded with a secondary stimulus. Neonatal mouse exposures from PND 4–7 and 10–13 increased GFAP in the hippocampus, corpus callosum, and anterior commissure in females, while males responded with decreased GFAP [58]. These measurements were made immediately following exposure, at two weeks. IBA1 was upregulated in the hippocampus and corpus callosum for males, at eight weeks and 9 months, respectively [53,58]. Females showed no change for IBA1. Adult mice of several genotypes showed brain inflammatory responses, with induction of IL-1a and TNFa, and activation of microglia and astrocytes [13].

Inhalation of prenatal diesel exhaust, as well as oropharyngeal aspiration of DEP, did not elicit any changes in GFAP or IBA1 in mice, examined at six months of age [48,51]. However a high fat diet (HFD) starting at 4 months and lasting for six weeks, along with the prenatal DE exposure, increased IBA1, but not GFAP [48]. IBA1 was increased in hypothalamus, dentate gyrus, amygdala, and the CA1 of the hippocampus for males [48]. Females showed changes only in the hypothalamus and dentate gyrus [48]. This upregulation of IBA1 supports the hypothesis of air pollution exposure during gestation as an enhancer for later life environmental insults: the DE+HFD group responded more than either treatment alone, with changes in brain regions that did not change with only one of the treatments (Table 3). The two-hit hypothesis postulates that the first insult primes the system [48,60]. While inflammation may not be activated by a single insult, the second inflammatory challenge may cause a disproportionately larger response.

### 4.7. Sex differences

Sex differences are apparent in rodent responses to prenatal exposure, with greater male vulnerability observed. For open field activity, only male mice showed deficits [48]. For the tail suspension test, a measure of depressive behavior, only males were responsive, with no effect for females [54]. Further sex differences were shown in secondary treatments (Table 3). Release of inflammatory cytokines is profoundly affected by sex, a trend even more pronounced with the addition of a secondary insult. When prenatal diesel exposure is combined with either six weeks HFD, or nest restriction from gestational day (GD) 14–19, male mice show significant effects in numerous cytokines and chemokines, while females show no changes [51,66]. The combination of prenatal diesel exposure and adult high fat diet increased serum insulin, insulin resistance, and IL-1b, again only in male mice [51]. Brain inflammatory proteins CD11b, TLR4, and CXC3CR1 were increased only in males [51]. Likewise, only males had increased peripheral macrophage infiltration in the hypothalamus [51]. Furthermore, for nest restriction paired with diesel exposure, only males showed decreased contextual fear recall, and changes in brain TLR4, caspase-1, IL-1b, and IL-10 [28]. Finally, in the combination of PND 4–7, 10–13, and 55–60 CAPS exposure, only males showed increased IBA1 staining in the corpus callosum [53]. These experimental findings demonstrate greater male vulnerability to pollution exposure, especially when combined with a second insult.

### 4.8. Nanoparticles in utero

One hypothesis for the mechanism underlying air pollution's physiological effects is unique to the nanoscale particulate matter component. It is possible that the particles might be able to cross the placental barrier to directly interact with the fetus, which develops a blood-brain barrier by gestational day 16 [67]. There is experimental evidence for transplacental transfer of nano-size PM. Titanium dioxide nanoparticles (25–70 nm dia) subcutaneously delivered to pregnant mice on GD 3, 7, 10 and 14, were detected in male brains and testes six weeks postnatally [68]. Thus, model nanoparticles can cross both the maternal placenta and the blood-brain barrier of the developing fetus. *Ex vivo* models with human placenta and polystyrene beads show strong size dependency, with 50 and 80 nm beads rapidly crossing the placenta, possibly by simple diffusion; larger beads > 240 nm do not cross [69]. However, this *ex vivo* model does not represent potential modification of PM by proteins and lipids, which create a bio-corona, altering the movement of the nanoparticles [70]. Engineered nanoparticles of this size show 1000-fold range of translocation to the brain (0.00006% to 0.03%) [63,71]. Although particles may cross secondary barriers (placenta, blood-brain), their inhalation or ingestion does not necessarily allow transport to these secondary barriers. This is of relevance because most experiments inject the particles into the animal, bypassing the lungs. The small size of nanoparticles is important, as particles < 34 nm rapidly translocate from lung to mediastinal lymph node [72]. Notable, negatively charged particles accumulated in secondary organs more than positively charged particles [73].

The placenta may be more vulnerable to nanoparticle entry later in gestation, when the placental wall has thinned and is more vascularized, but also early in gestation before the placenta is fully formed [50]. The period after the placenta is formed, but before maternal-fetal circulatory systems are fully developed, could be less vulnerable to pollution exposure, due to minimal blood flow to the fetus. Nanoparticles may even cause fetal damage without penetrating the placenta, e.g. *in vitro,* nanoparticles can cause DNA damage even when they do not cross a cell barrier [74]. We note that these are not exclusionary hypotheses, and both may potentially be occurring.

### 4.9. Protective measures

In the urban realities of 21^st^ Century populations, it is not possible to prevent prenatal exposure to TRP by restricting household or school proximity to roadways. Thus, we must consider other means to limit detrimental effects of prenatal TRP exposure. Diet optimization may be a pragmatic approach. As a precedent, higher maternal consumption of fruits and vegetables was associated with better mental development in exposure to benzene and NO_2_ [36]. Supplementation with anti-oxidants, e.g. the omega-3 fatty acid, docosahexaenoic acid, may blunt TRP induced oxidative stress [75]. While such interventions could attenuate effects, real solutions must come from technical advancements to lower the use of fossil fuels. Combustion engines can be made more efficient, while petroleum itself can be replaced by methanol, which produces fewer particles during combustion [76].

## 5. Conclusions

Traffic related air pollution is correlated with numerous detrimental health outcomes: increased cardiovascular mortality from adulthood exposures, and low birth weight and cognitive disorders from gestational exposure. These epidemiological observations are largely verified in animal models. The field is emergent with only a handful of labs worldwide studying animal models. There are huge gaps in the understanding of how TRP affects the brain. Specifically, neuronal changes, both in protein and morphology, and potential epigenetic modifications, are lacking. Another challenge comes from the different experimental paradigms between labs, particularly the source of PM and delivery regimen. Relatively few observations have been corroborated across labs. Even so, there is a clear trend for numerous adverse cognitive effects from pollution exposure during development, and future studies hold many promises.

## Figures and Tables

**Table 1:** Air pollution exposure sources

Exposure study	Methods	Particulate matter size and composition	Volatiles	Non-tailpipe pollution	Secondary aerosols
Diesel exhaust					

Direct from diesel engine Bolton et al. 2012 [48]	Auten et al. 2012 [47]	18-200 μm; extractable organic matter, 39.8%	Yes	No	No
Sugamata et al. 2006 [77], Fujimoto et al. 2005 [46], Yokota et al. 2009 [39], Yokota et al. 2013 [57], Suzuki et al. 2010 [62]	Umezawa et al. 2011 [78]	All size particles	CO, 2.67 ppm; NO_2_, 0.23 ppm, SO_2_, < 0.01 ppm	No	No
Diesel exhaust particles Bolton et al. 2013 [67], 2014* [51]	Auten et al. 2012 [47] Hougaard et al. 2008** [40]	18-200 μm	No	No	No

Traffic-related air pollution					

Ambient Air Wei et al., in prep. [49]		All size particles	Yes	Yes	Yes
Concentrated Ambient Particle System (CAPS)	Allen et al. 2013 [79], 2014a [53], 2014b [58]	< 100 nm dia.	Yes	Yes	Yes
Filter-trapped nano-sized PM (nPM) Davis et al. 2013 [54]	Morgan et al. 2011 [43]	< 200 nm dia. Filter-bound, re-aerosolized.	No	Yes	Yes

Non-tailpipe pollution includes brake dust, tire erosion, and roadway dust. Secondary aerosols arise from alterations of particulate matter by temperature, sunlight, humidity, ozone etc.

* Particles delivered by oropharyngeal introduction.

** Diesel exhaust particles reaerosolized for inhalation.

**Table 2:** Prenatal exposure: body weight, behavior, and cell-molecular responses

Study	Exposure protocol	Weight, gross changes	Behavior	Cell-molecular changes
Diesel exhaust direct				
Bolton et al 2012 [48], 2013 [66]; GD 18 (♂ + ♀)	0.5 or 2.0 mg/m^3^ 4 h/d; GD 7–17	Weight at 4 m: ♂—1.1 ♀—NC	Bolton 2012 [48]: Open field activity at 4 m: ♂—0.7 ♀—NC Bolton 2013 [66]: NC in forced swim	CCL2/MCP-1—3.5 CX3CL1/fractaline—1.5
Auten et al 2012 [47] GD 18 (♂ + ♀)	0.5 or 2.0 mg/m^3^ 4 h/d; GD 9–17	Weight at 4 w: 0.5 mg/m^3^ (♂ + ♀) 0.9 2.0 mg/m^3^ (♂ + ♀) NC		eotaxin: 4 KC: 6 RANTES: 10 +
Fujimoto et al 2005 [46]; GD 14	0.3, 1.0, or 3.0 mg/m^3^ 12 h/d; GD 2–13	↑ placental weight *(*♂, ♀—1.0 mg/m^3^) ↓ fetal weight *(*♂, ♀—3.0 mg/m^3^)		
Yokota et al 2013 [57] ♂ only- behavior at 5 w	1.0 mg/m^3^ 8 h/d; GD 2–17		↓ Retention time on rotating rod Cliff avoidance latency to jump 0.68	
Yokota et al 2009 [39] 5 w-behavior	1.0 mg/m^3^ 8 h/d; GD 2–17		Spontaneous motor activity: ♂—0.83	
Suzuki et al 2010 [62] 5 w	0.171 mg/m^3^, 8 h/d, 5 d/w GD 2–16		↓ Spontaneous locomotor activity	
DEP				
Hougaard et al 2008 [40] —19 w (♂ + ♀)	19 mg/m^3^ DEP 1 h/d; GD 9–19	Weight at 4 w: 0.9	No effect in the Morris water maze	No indication of any DNA damage, nor inflammation
CAPS				
Allen et al 2013 [79], Allen et al 2014a [53]	15–240 μg/m^3^ 4 h/d ; PND 4–7		♂ ↓ Response rates for FI60 (6 mo)	
PND 60	& 10–13 4 h/d; PND 56–60		Novel object performance (6 mo): ♂—0.5, ♀—0.8	
Allen et al 2014b [58] 2 w or 8 w	200,000 particles/cm^3^ 96 μg/m^3^ 4 h/d PND 4–7 & 10–13	Lateral ventricle size: PND14: ♂ 3.2 PND55: ♂ 1.8		GFAP PND 14: Hipp: ♂—0.5, ♀—1.9 CC: ♂—NC, ♀—1.5 IBA1 PND 55: Hipp: ♂—NC, ♀—NC AC: ♂—1.3, ♀—NC
Filter-trapped nPM				
Davis et al 2013 [54] —8 m	350 μg/m^3^ 5 h/d 3 d/w, 10 w		Tail suspension immobility 8 m: ♂—2.6, ♀—NC	PD3—JNK1 (♂, ♀) 0.7 Hipp GLU—NC

Abbreviations: AC, Anterior commissure; CAPS, Concentrated Ambient Particle System; CC, Corpus callosum; CCL2, Chemokine (C-C motif) ligand 2; CX3CL1, Chemokine (C-X3-C motif) ligand 1; DEP, diesel exhaust particles; FI60, Fixed interval reward 60 sec; GFAP, Glial fibrillary acidic protein; GD, Gestation day; GLU, Glutamate; Hipp, Hippocampus; IBA1, ionized calcium binding adaptor molecule 1; JNK-1, c-Jun N-terminal kinase 1; KC, keratinocyte chemoattractant; m, months; MCP-1, monocyte chemotactic protein 1; NC, no change; RANTES, regulated on activation, normal T cell expressed and secreted; w, weeks.

**Table 3:** Shared responses to traffic related air pollution across multiple experiments

Shared responses		No Change
Body weight	Auten 2012 [47] ↓ 4 w (DE), Bolton 2012 [48] ↑ 5 m (DE), Fujimoto 2005 [46] ↓ GD 14 (DE), Hougaard 2008 [40] ↓ 4 w (DEP), Sugamata 2006 [77] ↓ 4 w (DE), Umezawa 2011 [78] ↓ 8 w (DE), Wei unpub↓ [49] 2 w & 3 w (Beijing Air)	Allen 2014a [53], Bolton 2014 [51], Davis 2013 [54], Hougaard 2009 [41], Suzuki 2010 [62], Yokata 2013 [57]
Spontaneous locomotor activity	Bolton 2012 [48] ↓ 5 m (DE), Hougaard 2009 [41] ↓ 8 w (DE), Suzuki 2010 [62] ↓ 5 w (DE), Yokata 2009 [57] ↓ 5 w (DE)	Allen 2013 [79], Allen 2014a [53], Bolton 2014 [51], Davis 2013 [54]
Cortex-dopaminergic	Allen 2014a [53] ↓ 9 m (PND 55 CAPS), Allen 2014b [58] ↑ 2 w & 8 w (PND 14 CAPS), Suzuki 2010 [62] ↑ 5 w (DE), Yokata 2013 [57] ↓ 3 w (DE)	
Microglial activation	Allen 2014a [53] ↑ 9 m (PND14 + 55 CAPS), Allen 2014b [58] ↑ 8 w (CAPS), Bolton 2012 [48] ↑ GD 18 (DE), Bolton 2014 [51] ↑ 6 w (DEP + HFD)	Bolton 2013 [66]

Abbreviations: CAPS, Concentrated Ambient Particle System; DE, diesel exhaust; DEP, diesel exhaust particles; HFD, high fat diet; PND, Postnatal day

**Table 4:** Postnatal exposure, secondary manipulations.

Study (secondary treatment)	Behavior	Weight	Serum	Brain cellular	Brain subcellular
Diesel exhaust-direct					
Bolton et al 2012 [48]— 6 m DE + HFD HFD—45% fat, beginning 4 m, for 6 w		Weight ♂—NC ♀—4.4	Leptin: ♂—NC ♀—1.7 Insulin: ♂—6.4 ♀—NC	IBA1: Hypothalamus: ♂—1.7, ♀—1.3 Dentate gyrus: ♂—1.4, ♀—1.5 Amygdala: ♂—1.3, ♀—NC Hipp CA1: ♂—1.3, ♀—NC	
Bolton et al 2013 [66] DEP + Nest Restriction DEP: 50 μg DEP every 3 d, from GD 2-17 NR: E 14-19	Contextual fear recall: ♂—0.4 ♀—NC Anxiety behavior: ♂—1.6 ♀—1.2	No change			GD18 TLR4: ♂—1.3, ♀—NC Casp-1: ♂—1.4, ♀—NC IL-1b: ♂—NC ♀—NC Il-10: ♂—0.8, ♀—1.4
Bolton et al 2014 [51] DEP + High Fat Diet DEP: 50 μg DEP every 3 days, from GD 2-17 HFD: 45% Fat, beginning 4 m, for 9 w	Anxiety: (elevated zero maze) ♂—1.1 ♀—NC	Weight: ♂—1.3 ♀—NC	Insulin: ♂—1.8 ♀—NC	↓ peripheral macrophage infiltration in hypothalamus (♂1.2) GFAP Hipp NC	Hipp: CD11b: ♂—1.6, ♀—NC TLR4: ♂—1.2, ♀—NC CXC3CR1: ♂—1.2, ♀—NC
CAPS					
Allen et al 2013 [79]— Allen et al 2014a [53]— 9 m CAPS: PND 14 and/or PND 55	Waiting for reward behavior: ♂—**↓**			Corpus Callosum: IBA1: ♂—3.4, ♀—NC, C/A ♂—3.3, ♀—NC, A/C	Hipp: GLU: ♂—1.6, ♀—0.6, C/C DOPAC: ♂—NC, ♀—1.2, C/C

Abbreviations: A/C, Air/CAPS; C/A, CAPS/Air; CAPS, Concentrated air particle system; Casp-1, Caspase-1; CD11b, Cluster of differentiation 11 beta; CXC3CR1, Chemokine (C-X3-C motif) receptor 1; DOPAC, 3,4-Dihydroxyphenylacetic acid; GFAP, Glial fibrillary acidic protein; GLU, Glutamate; IBA1, Ionized calcium binding adaptor molecule 1; IL-1b, Interleukin-1 beta; IL-10, Interleukin 10; IR, Insulin resistance; HFD, High fat diet; Hipp CA1, Hippocampus, cornu Ammonis area 1; NC, No change; NR, Nest restriction; TLR4, Toll-like receptor 4.

**Table 5:** Neurotransmitter responses

Experiment	Yokota 2013 [57]		Suzuki 2010 [62]	Allen 2014a [53]		Allen 2014b [58]			

Exposure Condition	Prenatal exposure		Prenatal exposure	Neonatal Exposure		Neonatal Exposure			
Age	♂ 3 w	♂ 6 w	♂ 5 w	♂ 8 w	♀ 8 w	♂ 2 w	♀ 2 w	♂ 8 w	♀ 8 w

Cortex									

DA	0.6	NC	1.6	NC	NC				
DOPAC	NC	NC	2.0	NC	NC				
DOPAC:DA				NC	NC	NC		NC	
HVA	NC	NC	NC						
3-MT	NC	NC	1.5						
GABA				NC	NC				
GLU				NC	NC				
NE			2.0	NC	NC	NC	NC	1.2	0.9
MHPG			1.4						
NM			1.5						
5-HT	NC	1.3							
5-HIAA	NC	NC							

Hippocampus									

DA			NC						
DOPAC					NC	NC		NC	
DOPAC:DA							1.4		NC
HVA			0.2	NC	NC				
3-MT			NC						
GABA				NC	NC		NC		NC
NE			NC	NC	NC				
MHPG			0.7						
NM			NC						
5-HT	NC	NC					NC		1.3
5-HIAA	NC	NC							

Midbrain									

DA							NC		NC
DOPAC:DA						NC	0.7	NC	NC
HVA			0.7	NC	NC		NC		NC
3-MT			0.7						
NE			NC	NC	NC				
MHPG			0.6						
NM			NC						
5-HIAA				NC	NC				

Striatum									

DA			NC	0.8	NC				
DOPAC			NC	NC	NC				
DOPAC:DA				NC	NC				
HVA			NC	0.9	NC				
3-MT			NC						
NE			NC	NC	NC				
MHPG			0.8						
5-HT	NC	NC		NC	NC				
5-HIAA	NC	NC		0.7	NC				

Cerebellum									

DA			NC						
HVA			NC						
NE			NC						
MHPG	0.8	0.8	0.6						
NM	NC	0.6	NC						
5-HT	NC	NC							
5-HIAA	NC	NC							

Hypothalamus									

DA				NC	NC				
DOPAC				NC	NC				
DOPAC:DA				NC	NC				
NE	NC	1.2		0.7	NC				
HVA				NC	NC				
5-HT	NC	NC							
5-HIAA	NC	1.4							

Amygdala									

DA	1.5	NC							
DOPAC	1.4	1.3							
HVA	1.4	NC							
3-MT	1.4	NC							
5-HT	1.4	NC							
5-HIAA	1.3	NC							

Fold changes (approximated from figures when not given). Abbreviations: DA, dopamine; D OPAC, 3,4-dihydroxyphenylacetic acid (DA catabolite); GABA, gamma-aminobutyric acid; GLU, glutamate; HVA, homovanillic acid (DA catabolite); MHPG, 4-hydroxy-3-methoxyphenylglycol (NE catabolite); NC, No change; NE, norepinephrine; NM, normetanephrine (NE catabolite); 3-MT, 3-methoxytyramine (NE catabolite); 5-HIAA, 5-hydroxyindoleacetic acid (serotonin catabolite); 5-HT, serotonin.

**Table 6:** Cytokine changes

	Allen 2014b [58]				Bolton 2012 [48]

	♂ 2 w	♀ 2 w	♂ 8 w	♀ 8 w	♂ + ♀ GD 18
Cortex					

IL-6				0.5	
IL-1b	NC		NC		

Hippocampus					

IL-6	NC		NC		
IL-1b	NC		NC		

Midbrain					

IL-6		NC		2.1	
IL-1b	0.5	NC	1.4	NC	
TNF-a	0.5	NC	1.5	NC	

Striatum					

IL-6		0.4		NC	
IL-1b	NC		NC		
TNF-a	NC	0.7	NC	NC	

Whole Brain IL-6					3.7

IL-1b					5.5
TNF-a					
IL-10					3.5

Abbreviations: GD, Gestational day: NC: No change; PND: Postnatal day.
